# Assessment of the insecticidal activity of afoxolaner against *Aedes aegypti* in dogs treated with NexGard^®^

**DOI:** 10.1051/parasite/2017042

**Published:** 2017-10-23

**Authors:** Julian Liebenberg, Josephus Fourie, Wilfried Lebon, Diane Larsen, Lenaïg Halos, Frédéric Beugnet

**Affiliations:** 1 Clinvet International (Pty) Ltd, PO Box 11186, 9321 Universitas South Africa; 2 Boehringer Ingelheim Animal Health, 29 avenue Tony Garnier, 69007 Lyon France

**Keywords:** *Aedes aegypti*, insecticide, afoxolaner, NexGard^®^, dog

## Abstract

Twelve healthy dogs were studied in this parallel group, blinded, randomised, and negative controlled efficacy study. On Day -1, the 12 dogs included were ranked within sex in descending order of individual pre-treatment (Day -5) fed mosquito counts and randomly allocated by blocks of two dogs to the untreated control group or the afoxolaner-treated group. NexGard^®^ (Merial, now part of Boehringer Ingelheim Animal Health) was administered orally on Day 0 in accordance with the European label instructions. On Days 1, 7, 14, 21 and 28, all dogs were exposed for a duration of 1 hour to 50 ± 5 unfed *Aedes aegypti* females. After each exposure, mosquitoes were collected after 1 hour and assessed for viability during collection and at 24 ± 2 hours. The arithmetic (and geometric) mean values of live fed mosquito counts at 24 hours after the exposure periods for the negative control group ranged from 33.7 (32.3) to 49.8 (49.7), indicating that this was a vigorous mosquito strain. There was no significant difference between control and treated groups in the number of live and fed mosquitoes at each 1 hour post-exposure collection time. Based on arithmetic and geometric mean values at 24 hours after each exposure, significantly fewer live fed mosquitoes were recorded in the treated group, compared to the negative control group, throughout the study (*p* < 0.001). The afoxolaner insecticidal efficacy against *A. aegypti* varied from 98% (Day 2) to 75.3% (Day 29) based on arithmetic means, and 98.7% (Day 2) to 89.8% (Day 29) based on geometric means.

## Abstract (fr)

Douze chiens en bonne santé ont été étudiés dans cette étude d'efficacité en aveugle, en groupes parallèles et avec contrôles négatifs. Au jour -1, les 12 chiens inclus ont été classés par sexe par ordre décroissant de prétraitement individuel (jour -5) de comptage de moustiques nourris et répartis au hasard par blocs de deux chiens, en groupe témoin non traité et en groupe traité par afoxolaner. Du NexGard^®^ (Merial, maintenant un membre de Boehringer Ingelheim Animal Health) a été administré par voie orale au jour 0 conformément aux instructions dela notice européenne. Aux jours 1, 7, 14, 21 et 28, tous les chiens ont été exposés pendant une durée de 1 heure à 50 ± 5 *Aedes aegypti* femelles à jeun. Une heure après chaque exposition, les moustiques ont été recueillis et leur viabilité a été évaluée à la collecte et après 24 ± 2 heures. Les valeurs moyennes arithmétiques (et géométriques) du nombre de moustiques vivants et nourris 24 heures après les périodes d'exposition pour le groupe témoin négatif variaient de 33,7 (32,3) à 49,8 (49,7), ce qui indique une bonne viabilité de la souche de moustiques. Il n'y avait pas de différence significative entre les groupes témoins et les groupes traités dans le nombre de moustiques vivants et nourris à chacune des collectes à 1 heure post-exposition. Sur la base des valeurs moyennes géométriques et arithmétiques, à 24 heures après chaque exposition, des nombres significativement plus petits de moustiques nourris et vivants ont été enregistrés dans le groupe traité par rapport au groupe témoin non traité pendant toute l'étude (*p* < 0,001). L'efficacité insecticide de l'afoxolaner contre *A. aegypti* variait de 98 % (jour 2) à 75,3 % (jour 29) sur la base de moyennes arithmétiques, 98,7 % (jour 2) à 89,8 % (jour 29) sur la base de moyennes géométriques.

## Introduction

Recently, a new class of insecticides/acaricides, the isoxazolines, have demonstrated very good efficacy against fleas and ticks [19]. Afoxolaner is an isoxazoline administered monthly to protect dogs against fleas and ticks (NexGard^®^, Merial, now part of Boehringer Ingelheim Animal Health) [2,3,8,10]. It is administered at a minimum dose of 2.5 mg/kg. Recent studies have demonstrated its activity against other arthropods, including *Demodex canis*, the agent of canine demodicosis, *Sarcoptes scabiei* var. *canis* and *S. scabiei var. suis*, the agent of sarcoptic mange in dogs and swine, respectively, as well as *Otodectes cynotis*, the agent of ear mange in dogs and cats [1,4,5].

After oral administration, afoxolaner is absorbed quickly, with peak plasma levels (Cmax) reached between 2 to 4 hours after administration [14,15]. Plasma protein binding is more than 99%, which explains the long half-life, 10–14 days on average [14,15]. Due to its strong binding to plasma proteins, its activity is systemic and exposure is related to the ingestion of blood or inflammatory fluids by the biting insect.

In addition to its activity against well-known blood-feeding ectoparasites like fleas and ticks, or resident ectoparasites like *Demodex, Sarcoptes,* and *Otodectes*, it is probable that afoxolaner would also have a certain level of insecticidal activity against other blood-feeding arthropods like mosquitoes. Insecticidal efficacy following a blood meal might not prevent pathogen transmission from the female mosquito, but it could have a further effect by killing the mosquitoes before a new bite, and/or by reducing the mosquito population in a restricted area like a household where treated dogs are living. It could therefore have an indirect action on the rate of vector-borne pathogen transmission within the household.

*Aedes aegypti* mosquitoes are endemic in tropical areas around the globe, but have expanded into sub-tropical areas and even some warm temperate locations, although the species seems less adaptable to temperate climate than *Aedes albopictus* [13]. It is now found in many parts of the world including South and Central America, the southern USA, Africa, India, tropical islands, South-East Asia, Northern Australia, and sporadically in the Mediterranean zone [13]. *A. aegypti* is a major vector of several diseases of animals and/or humans, e.g. heartworm disease due to *Dirofilaria immitis* in dogs, equine encephalitis viruses, West Nile virus, Dengue virus, Chikungunya virus, Zika virus, and yellow fever virus [9,12]. The objective of this study was to assess the insecticidal activity that afoxolaner may have against *A. aegypti* mosquitoes.

## Materials and methods

The design and conditions of this study were approved by the South African and ClinVet animal welfare ethics committees, and were performed in accordance with the Good Clinical Practices of the European Agency for the Evaluation of Veterinary Medicinal Products (CVMP/VICH GL9, July 2000; CVMP/VICH GL19, July 2001). This study was a parallel group, blinded, randomised, negative controlled efficacy study. It was conducted with two groups of six dogs each.

Male and female dogs were included in the study if they had been acclimatised to the study conditions for at least 8 days; they were clinically healthy as verified by a veterinarian on Day -8; they were ≥ 6 months at the time of inclusion (Day -1); females were not pregnant; they had not been treated with a long-acting topical or systemic acaricide/insecticide during the 12 weeks preceding Day 0.

The animals were kept individually in cages and no physical contact between dogs was possible. However, animals still had visual and auditory contact with conspecifics. During the acclimatisation period (Day -8 to Day -1), an initial *A. aegypti* mosquito challenge was performed on Day -5 to evaluate the receptivity of each dog to experimental infestation and for random allocation of the dogs to the study groups. The 12 dogs included in the study were randomly allocated to two groups (untreated control group and afoxolaner-treated group), based on total counts of fed mosquitoes 1 hour after the initial challenge. In addition, veterinary clinical examination was performed on Day -8 for enrolment purposes, and weighing of all dogs was performed on Day -1 for appropriate dose determination. All the dogs were observed daily from Day -8 to Day 28 for their general health.

On Day 0, all dogs assigned to the treated group received afoxolaner. The dogs were treated orally with NexGard^®^ (2.27% w/w afoxolaner chewable tablets) in accordance with European label instructions [8]. All dogs weighed from 10 to 25 kg and were treated with a chewable tablet containing 68 mg of afoxolaner. The dogs were observed hourly for 4 hours after administration to detect possible adverse reactions.

Dogs were challenged with 50 ± 5 *Aedes aegypti* unfed female mosquitoes on Day -5 for randomisation purposes, and then on Days 1, 7, 14, 21 and 28 to assess insecticidal activity. Mosquitoes were assessed for viability and feeding status during collection 1 hour after exposure and on Days 2, 8, 15, 22 and 29 (24 hours after exposure).

To perform the mosquito challenge, the dogs were sedated using medetomidine (Domitor^®^, Zoetis), and placed into a mosquito proof net (dimensions: 81 cm × 58 cm × 58 cm). The whole body of the dog was thus exposed to the mosquito challenge. The mosquito net used allowed both exposure of dogs to the parasites and collection of parasites after the challenge, without mosquitoes escaping during the process. A Clinvet laboratory-bred strain of *A. aegypti* of US origin was used in the infestation challenges.

Food was removed at least two hours prior to sedation of animals or animals were fasted overnight if required by scheduling constraints. The 50 ± 5 female mosquitoes were released into the net and they were carefully vacuumed after 1 hour.

At the end of the exposure period, atipamezole (Antisedan^®^, Zoetis) was used to reverse the effects of the sedation in dogs.

One hour after challenge, the mosquitoes were collected using an aspirator and they were then assessed as live, fed or unfed, moribund or dead. Mosquitoes were classified as live if they exhibited normal behaviour and were capable of coordinated locomotion and flight upon external stimuli. Mosquitoes were classified as moribund if they were only capable of some movement, but exhibit abnormal, obviously impaired behaviour, and were not capable of coordinated locomotion or flight upon external stimuli.

Prior feeding by dead mosquitoes was assessed following the collection, by placing the dead mosquito on tissue paper and squashing the abdomen with a spatula or similar suitable object to assess if a blood meal was taken.

Live and moribund mosquitoes were incubated in suitable containers at 24.3 °C to 28.1 °C for 24 hours (± 2 hours). During this period the mosquitoes had access to a 10% sucrose solution, or a suitable alternative. The mosquitoes were again assessed for viability following the 24-hour (± 2 hours) incubation period and then assessed for feeding as described above. All live and moribund mosquitoes were immobilised in a freezer prior to the feeding assessments.

The dead mosquito counts observed after each challenge are the sum of the dead mosquitoes counted at 1 hour (Table 1) and the dead mosquitoes counted at 24 hours (Table 2).

**Table 1:** Comparison of body weights between dogs and mosquito counts obtained 1h after exposure at Day -5 for allocation purposes.

	Day	Statistic	Control dogs	Afoxolaner-treated dogs
Body weight (kg)	Day -1	n	6	6
*p*-value: 0.8655		Mean	16.37	16.03
		SD	2.467	3.999
		Median	16.00	14.70
		Minimum	13.8	12.4
		Maximum	19.2	23.6
Mosquito count 1h after exposure	Day -5	n	6	6
*p*-value: 0.5008		Mean	53.3	54.0
		SD	2.25	0.63
		GeoMean	53.3	54.0
		Median	53.0	54.0
		Minimum	50	53
		Maximum	57	55

*p*-value: One-way ANOVA with a treatment effect.

**Table 2:** Mosquito status assessment during collection at 1h post-challenge.

		Day 1			Day 7			Day 14			Day 21			Day 28		
						
Group	Dog ID															
		Live	Moribund	Dead	Live	Moribund	Dead	Live	Moribund	Dead	Live	Moribund	Dead	Live	Moribund	Dead
Control dogs	2A9 928	53	0	0	49	0	2	53	0	3	52	0	2	49	0	2
	2AA 13E	51	0	3	45	0	4	53	0	2	55	0	1	62	0	1
	5A6 23A	54	0	0	48	0	2	35	0	13	50	0	2	56	0	1
	5BF 5E2	54	0	1	46	0	1	51	0	2	47	0	3	50	0	2
	5CC 399	52	0	2	48	0	0	53	0	2	51	0	0	51	0	1
	DF7 F0E	52	0	0	45	0	5	51	0	4	39	0	11	57	0	0
Arithmetic means		52.7	0.0	1.0	46.8	0.0	2.3	49.3	0.0	4.3	49.0	0.0	3.2	54.2	0.0	1.2
% Dead mosquitoes				1.86%			4.68%			8.02%			6.13%			2.16%
Afoxolaner-treated dogs	284 46C	51	0	1	50	0	3	53	0	2	50	0	6	54	0	4
	289 EAA	55	0	0	49	0	0	49	0	0	51	0	0	48	0	0
	2AC 6FC	57	0	0	37	0	7	45	0	4	50	0	2	54	0	0
	5CE 690	31	0	2	46	0	0	49	0	5	45	0	2	48	0	4
	B2C 501	44	0	2	51	0	1	52	0	2	50	0	2	55	0	1
	E19 6E0	51	0	1	49	0	1	54	0	1	53	0	3	50	0	0
Arithmetic means		48.2	0.0	1.0	47.0	0.0	2.0	50.3	0.0	2.3	49.8	0.0	2.5	51.5	0.0	1.5
% Dead mosquitoes				2.03%			4.08%			4.37%			4.78%			2.83%

Insecticidal activity calculations were based on both arithmetic and geometric mean values. Geometric mean efficacy calculations were based on the geometric mean values of the mosquito (count + 1) data. One (1) was subsequently subtracted from the result to obtain a meaningful value for the geometric mean of each group.

The primary efficacy of afoxolaner against *A. aegypti* mosquitoes was calculated using the total live fed mosquitoes at 24 hours after each mosquito challenge, according to the formula below:

Insecticidal efficacy (%) against mosquitoes = 100 x (Mc − Mt)/Mc, where:

Mc = mean number of live fed mosquitoes in the control group at 24 hours after challenge;

Mt = mean number of live fed mosquitoes in the treated group 24 hours after challenge.

The groups were compared using an ANOVA (Proc GLM procedure in SAS) with a treatment effect on both untransformed and logarithmic transformed mosquito (count + 1) data. SAS Version 9.3 TS Level 1M2 was used for all the statistical analyses.

## Results

The weight of dogs varied from 13.8 to 19.2 kg in the control group (mean = 16.37 kg) and from 12.4 to 23.6 kg (mean value 16.02 kg) in the treated group. No statistically significant differences were recorded between the pre-treatment fed mosquito counts at Day -5 (*p* = 0.5008) nor the body weights (*p* = 0.8655) of the dogs in the two groups, which indicated homogeneity between the dogs included in each group (Table 1).

No adverse events were recorded after treatment or during the study duration [6].

The live, moribund and dead status of the mosquitoes was assessed at all time-points (Tables 2 and 3). The collection of live mosquitoes at 1 hour post-exposure indicated a mortality of 1.86 to 8% during the contact time between dogs and mosquitoes (Table 2). There was no significant difference between the control group and the treated group in the numbers of live or dead mosquitoes at the end of the 1 hour exposure (Table 2). No moribund mosquitoes were observed at 1h.

**Table 3:** Counts of mosquitoes and assessment of their status 24h post-exposure (Days 2 8 and 15).

Group	Dog ID	Day 2							Day 8						Day 15					
				
		Live	Moribund	Dead	Fed (live + dead + moribund)		Unfed (live + dead + moribund)	Live Fed	Live	Moribund	Dead	Fed (live + dead + moribund)	Unfed (live + dead + moribund)	Live Fed	Live	Moribund	Dead	Fed (live + dead + moribund)	Unfed (live + dead + moribund)	Live Fed
Control dogs	2A9 928	53	0	0	53		0	53	48	0	3	46	5	45	53	0	3	52	4	49
	2AA 13E	51	0	3	47		7	46	42	0	7	45	4	42	50	0	5	38	17	35
	5A6 23A	53	0	1	53		1	52	43	0	7	47	3	41	22	8	18	31	17	20
	5BF 5E2	52	1	2	51		4	48	43	0	4	44	3	41	38	1	14	37	16	25
	5CC 399	51	0	3	49		5	47	44	0	4	45	3	41	53	0	2	32	23	32
	DF7 F0E	47	0	5	42		10	38	43	0	7	42	8	40	41	6	8	49	6	41
Arithmetic means		51.2	0.2	2.3	49.2		4.5	47.3	43.8	0..0	5.3	44.8	4.3	41.7	42.8	2.5	8.3	39.8	13.8	33.7
Afoxolaner-treated dogs	284 46C	14	3	35	38		14	0	10	0	43	41	12	0	3	18	34	50	5	0
	289 EAA	2	3	50	55		0	2	11	0	38	37	12	0	10	21	18	49	0	10
	2AC 6FC	0	8	49	57		0	0	3	0	41	35	9	0	11	7	31	33	16	0
	5CE 690	2	5	26	30		3	2	3	0	43	43	3	0	3	7	44	48	6	0
	B2C 501	6	2	38	37		9	0	0	0	52	51	1	0	21	23	10	44	10	11
	E19 6E0	1	5	46	52		0	1	1	0	49	49	1	0	14	27	14	41	14	2
Arithmetic means		4.2	4.3	40.7	44.8		4.3	0.8	4.7	0.0	44.3	42.7	6.3	0.0	10.3	17.2	25.2	44.2	8.5	3.8

Table 3 (continued). Counts of mosquitoes and assessment of their status 24h post-exposure (Days 22 and 29).													
Group	Animal ID	Day 22						Day 29					
			
		Live	Moribund	Dead	Fed (live + dead + moribund)	Unfed (live + dead + moribund)	Live Fed	Live	Moribund	Dead	Fed (live + dead + moribund)	Unfed (live + dead + moribund)	Live Fed
Control dogs	2A9 928	52	0	2	53	1	51	49	0	2	50	1	48
	2AA 13E	54	0	2	56	0	54	44	3	16	61	2	43
	5A6 23A	46	2	4	52	0	46	56	0	1	57	0	56
	5BF 5E2	32	10	8	46	4	30	50	0	2	51	1	49
	5CC 399	51	0	0	41	10	41	51	0	1	52	0	51
	DF7 F0E	28	5	17	47	3	26	57	0	0	52	5	52
Arithmetic means		43.8	2.8	5.5	49.2	3.0	41.3	51.2	0.5	3.7	53.8	1.5	49.8
Afoxolaner-treated dogs	284 46C	20	9	27	36	20	0	6	12	40	51	7	0
	289 EAA	15	1	35	33	18	0	5	4	39	46	2	3
	2AC 6FC	14	11	27	39	13	1	7	6	41	51	3	4
	5CE 690	9	21	17	39	8	1	2	10	40	50	2	2
	B2C 501	26	8	18	42	10	17	52	0	4	53	3	50
	E19 6E0	32	8	16	49	7	32	24	17	9	41	9	15
Arithmetic means		19.3	9.7	23.3	39.7	12.7	8.5	16.0	8.2	28.8	48.7	4.3	12.3

The arithmetic mean values of live fed mosquito counts at 24 hours after the challenge period for the negative control group ranged from 33.7 to 49.8, indicating that this was a vigorous mosquito strain. The insecticidal and acaricidal activities, following the European Medicine Agency guideline (EMA) [7] and the World Association for the Advancement of Veterinary Parasitology (WAAVP) [16], should be based on the comparison of the number of live arthropods collected from control and treated animals (Table 3). Arithmetic and geometric mean values of live fed mosquito counts and efficacies are summarised in Tables 3 and 4.

**Table 4:** Insecticidal efficacy based on live fed mosquitoes counted at 24 hours post-exposure.

	Control Group	Afoxolaner-treated Group		
			
Day	Arithmetic (Geometric) Mean	Arithmetic (Geometric) Mean	Percentage efficacy (based on geometric means)	ANOVA *p*-Value
Day 2	47.3 (47.1)	0.8 (0.6)	98.2 (98.7)	< 0.0001
Day 8	41.7 (41.6)	0.0 (0.0)	100.0 (100.0)	< 0.0001
Day 15	33.7 (32.3)	3.8 (1.7)	88.6 (94.7)	< 0.0001
Day 22	41.3 (40.0)	8.5 (2.7)	79.4 (93.4)	0.0005
Day 29	49.8 (49.7)	12.3 (5.0)	75.3 (89.8)	0.001

Based on both arithmetic and geometric mean values of live fed mosquitoes at 24 hours, significantly fewer live fed mosquitoes were recorded for the afoxolaner treated group compared to the negative control group, throughout the study (*p* < 0.001). Based on arithmetic and geometric mean values of live fed mosquitoes at 24 hours, the insecticidal efficacy of NexGard^®^ was 98.2% (98.7% based on geo mean), 100% (100%), 88.6% (94.7%), 79.4% (93.4%), and 75.3% (89.8%), on days 2, 8, 15, 22 and 29, respectively (Table 4).

## Discussion and conclusion

The study design classically used to assess repellency of insecticides after 1h of exposure of flying insects was used in this particular study to assess insecticidal activity after feeding [16]. Being systemic, afoxolaner binds to plasma proteins [15], and no repellent activity related to volatile molecules on the skin surface was expected. The number of fed mosquitoes observed at 1h was not different between the control and treated dogs (> 90% in both groups), thus confirming the absence of a repellent effect, which is assessed by the anti-feeding effect. No differences in the number of live and dead mosquitoes were observed at 1h collection, indicating that there was no immediate killing effect.

The insecticidal activity was high 24 hours after each exposure challenge, indicating that the *A. aegypti* mosquitoes ingested a lethal dose of afoxolaner during their blood meal. The blood meal of female *A. aegypti* takes only a few minutes (< 5 min) and the volume ingested is about 4-5 μL [9,11,17,18]. The study design based on 1 hour exposure to dogs allowed more than 90% of mosquitoes to feed and it is expected that the maximum proportion was reached in such a period. After a single administration at Day 0, the quantity of afoxolaner present in 4-5 μL of dog blood was enough to kill > 75% (> 89% in geometric mean) of the fed female mosquitoes throughout an entire month. There are currently no data on specific mosquito species sensitivity and these results should be confirmed in other important species such as *Culex pipiens* or *A. albopictus*.

Given the lack of anti-feeding effect, it is not expected that afoxolaner treatment in dogs would have a direct impact on the transmission of pathogenic agents by *A. aegypti* during the blood meal. Nevertheless, *A. aegypti* females need a blood meal every 2-3 days, and 48 hours is needed for oviposition [9,11,18]. This species is also unlikely to disperse and has restricted flight capacity, estimated to be less than 500 m [9,11]. The behaviour tends to be indoor. These biological aspects are in favour of a rapid decrease of the mosquito population biting treated dogs. A female would die after its blood meal and would not bite a second time; neither would the insect be able to lay eggs before dying. This hypothesis would need to be demonstrated under a simulated household environment, but the results of this study are in favour of an indirect protective effect in households where afoxolaner-treated dogs are living. Killing *Aedes* females before a new blood meal would reduce the rate of transmission of vector-borne pathogens, like heartworm in dogs.

## Conflict of interest

This clinical study was funded by Merial, now part of Boehringer Ingelheim Animal Health, 29 Avenue Tony Garnier, 69007 Lyon of which Frédéric Beugnet, Lénaïg Halos and Wilfried Lebon are employees.

ClinVet, of which Julian Liebenberg and Josephus Fourie are employees, is an independent South African Contract Research Organisation contracted to conduct the study.

All authors voluntarily publish this article and have no personal interest in these studies other than publishing the scientific findings that they have been involved in generating via planning, initiating, monitoring and conducting the investigations and analysing the results.

## Disclaimer

NEXGARD^®^ is a registered trademark of Merial SAS. All other brands are the property of their respective owners.

This document is provided for scientific purposes only. Any reference to a brand or a trademark herein is for informational purposes only and is not intended for a commercial purpose or to dilute the rights of the respective owner(s) of the brand(s) or trademark(s).

## References

[1] Beugnet F, de Vos C, Liebenberg J, Halos L, Larsen D, Fourie J. Efficacy of afoxolaner in a clinical field study in dogs naturally infested with *Sarcoptes scabiei*. 2016 Parasite, 23, 26. 27317462 10.1051/parasite/2016026 PMC4912682

[2] Beugnet F, Liebenberg J, Halos L. 2015 Comparative efficacy of two oral treatments for dogs containing either afoxolaner or fluralaner against *Rhipicephalus sanguineus sensu lato* and *Dermacentor reticulatus*. Veterinary Parasitology, 209, 142–145. 25716658 10.1016/j.vetpar.2015.02.002

[3] Beugnet F, Liebenberg J, Halos L. 2015 Comparative speed of efficacy against *Ctenocephalides felis* of two oral treatments for dogs containing either afoxolaner or fluralaner. Veterinary Parasitology, 207, 297–301. 25564276 10.1016/j.vetpar.2014.12.007

[4] Beugnet F., Halos L., Larsen D., de Vos C. Efficacy of oral afoxolaner for the treatment of canine generalised demodicosis. Parasite 2016, 23, 14. 27012161 10.1051/parasite/2016014 PMC4807374

[5] Carithers D, Crawford J, de Vos C, Lotriet A, Fourie J. Assessment of afoxolaner efficacy against Otodectes cynotis infestations of dogs. 2016 Parasites & Vectors, 9, 635. 27938395 10.1186/s13071-016-1924-4 PMC5148825

[6] Drag M, Saik J, Harriman J, Larsen D. 2014 Safety evaluation of orally administered afoxolaner in 8-week-old dogs. Veterinary Parasitology, 201, 198–203. 24629431 10.1016/j.vetpar.2014.02.022

[7] European Medicine Agency Committee for Medicinal Products for Veterinary Use. 2007. Guidelines for the Testing and Evaluation of the Efficacy of Antiparasitic Substances for the Treatment and Prevention of Tick and Flea Infestation in Dogs and Cats. *EMA Guideline No. EMEA/CVMP/EWP/005/2000-Rev2-2007*. London, 2007.

[8] European Medicines Agency. Nexgard^®^ summary of product characteristics. 2014 http://www.ema.europa.eu/docs/en_GB/document_library/EPAR_-_Product_Information/veterinary/002729/WC500164067.pdf

[9] Foster W.A., Walker E.D. 2009. Chapter 14: Mosquitoes, in Medical and Veterinary Entomology Second Edition, Edited by G.R. Mullen and L.A. Durden, Elsevier, London, 207-259.

[10] Halos L, Lebon W, Chalvet-Monfray K, Larsen D, Beugnet F. 2014 Immediate efficacy and persistent speed of kill of a novel oral formulation of afoxolaner (NexGard^®^) against induced infestations with Ixodes ricinus ticks. Parasites and Vectors, 7, 452. 25261196 10.1186/1756-3305-7-452 PMC4262065

[11] Kettle DS. 1995. Culicidae (Mosquitoes), in Medical and Veterinary Entomology, Ed CAB International, Oxon, UK, 109-151.

[12] Kock R.A. 2015 Vertebrate reservoirs and secondary epidemiological cycles of vector borne diseases. Revue Scientifique et Technique (International Office of Epizootics), 34, 151-153. 26470455 10.20506/rst.34.1.2351

[13] Kraemer M, Sinka ME, Duda KA, Mylne AQN, Shearer FM, Barker CM, Moore CG, Carvalho RG, Coelho GO, Van Bortel W, Hendrickx G, Schaffner F, Elyazar IRF, Teng HJ, Brady HJ, Messina JP, Pigott DM, Scott TW, Smith DL, Wint WGR, Golding N, Hay SI. 2015 The global distribution of the arbovirus vectors *Aedes aegypti* and *Ae. albopictus*. eLife, 4, e08347. 26126267 10.7554/eLife.08347 PMC4493616

[14] Letendre L, Harriman J, Drag M, Mullins A, Malinski T, Rhebein S. 2016 The intravenous and oral pharmacokinetics of afoxolaner and milbemycin oxime when used as a combination chewable parasiticide for dogs. Journal of Veterinary Pharmacology and Therapeutics, 40, 35–43 27604405 10.1111/jvp.12332

[15] Letendre L, Harriman J, Huang R, Kvaternick V, Drag M, Larsen DL. 2014 The intravenous and oral pharmacokinetics of afoxolaner, a novel isoxazoline, used as a monthly chewable antiparasitic for dogs. Veterinary Parasitology, 201, 190-197. 24685320 10.1016/j.vetpar.2014.02.021

[16] Marchiondo AA, Holdsworth PA, Fourie LJ, Rugg D, Hellmann K, Snyder DE, Dryden MW. 2013 World Association for the Advancement of Veterinary Parasitology (W.A.A.V.P.) second edition: guidelines for evaluating the efficacy of parasiticides for the treatment, prevention and control of flea and tick infestations on dogs and cats. Veterinary Parasitology, 194, 84-97. 23741753 10.1016/j.vetpar.2013.02.003

[17] Rodhain F. 2015 Insects as vectors: systematics and biology. Revue Scientifique et Technique (International Office of Epizootics), 34, 83-96. 26470450

[18] Russell RC, Otranto D, Wall R. 2013 Mosquitoes (Diptera: Culicidae), in The Encyclopedia of Medical and Veterinary Entomology, Ed CAB International, Wallingford, UK, 243-282.

[19] Shoop WL, Hartline EJ, Gould BR, Waddell ME, McDowell RG, Kinney JB, Lahm, GP, Long JK, Xu M, Wagerle T, Jones GS, Dietrich RF, Cordova D, Schroeder ME, Rhoades DF, Benner EA, Confalone PN. 2014 Discovery and mode of action of afoxolaner, a new isoxazoline parasiticide for dogs. Veterinary Parasitology, 201, 179-189. 24631502 10.1016/j.vetpar.2014.02.020

